# *RARB* genetic variants might contribute to the risk of chronic obstructive pulmonary disease based on a case-control study

**DOI:** 10.1080/07853890.2024.2445195

**Published:** 2024-12-26

**Authors:** Linhui Huang, Wenya Xu, Yihui Fu, Zehua Yang, Rubing Mo, Yipeng Ding, Tian Xie

**Affiliations:** a Department of Pulmonary and Critical Care Medicine, Hainan General Hospital, Hainan Affiliated Hospital of Hainan Medical University, Haikou, China; b Department of General Practice, Hainan General Hospital, Hainan Affiliated Hospital of Hainan Medical University, Haikou,China

**Keywords:** Chronic obstructive pulmonary disease, susceptibility, *RARB*, single nucleotide polymorphisms

## Abstract

**Background:**

Chronic obstructive pulmonary disease (COPD) is a progressive respiratory disease that severely impairs patients’ respiratory function and quality of life. RARB is involved in COPD progression by affecting inflammatory reactions, cell proliferation, and apoptosis. The impact of single nucleotide polymorphisms (SNPs) within RARB on COPD susceptibility remains unclear. Here, we aimed to evaluate the association between RARB SNPs and COPD risk.

**Methods:**

A total of 270 COPD patients and 271 healthy controls were enrolled. The MassARRAY iPLEX platform tested the genotype of the SNPs. The association was analyzed using logistic regression analysis. The false-positive report probability (FPRP) analysis was performed to validate the significant findings. The relationship between SNPs and RARB expression was evaluated using the GTEx database.

**Results:**

Our study found a significant association between rs6799734 and COPD susceptibility (OR 1.88, *p* = 0.008, *p* (FDR) = 0.047). The stratified analysis revealed that this association was particularly pronounced among individuals aged ≤ 71 years (OR 2.34, *p* = 0.011, *p* (FDR) = 0.045), males (OR 2.60, *p* = 0.002, p (FDR) = 0.013), those with a BMI ≥ 24 (OR 3.95, *p* = 0.018, *p* (FDR) = 0.108), and smokers (OR 2.48, *p* = 0.020, *p* (FDR) = 0.120). Additionally, rs1286641 and rs1881706 showed significant associations with COPD risk in females and smokers. These associations were further validated by FPRP analysis. Preliminary mechanism studies indicated that rs1286641 and rs1881706 were related to *RARB* expression.

**Conclusion:**

Our findings suggest a potential role of RARB SNPs in influencing COPD risk.

## Introduction

Chronic obstructive pulmonary disease (COPD) is a common respiratory disease, characterized by persistent respiratory symptoms and airflow obstruction. It is usually related to airway and/or alveolar abnormalities and is mainly caused by the abnormal inflammatory response of the lungs to harmful particles or gases [1]. The main clinical manifestations of COPD are chronic cough, expectoration, and dyspnea, which can lead to a significant decline in quality of life and an increase in the risk of death in severe cases [2]. The etiology of COPD is very complicated, including smoking, occupational exposure, air pollution, and genetic factors [3]. Smoking is the most important risk factor, but non-smokers may develop COPD due to genetic susceptibility, environmental exposure, and other factors [4]. Genetic factors play an important role in the occurrence and development of COPD. Studies have shown that genetic variation can affect the individual’s susceptibility to COPD, as well as the severity and progress rate of the disease [4,5]. For example, the polymorphism of some genes is related to the increased risk of COPD, such as the genetic variation of MIR1208 and MIR5708, which has been proven to be related to the susceptibility of COPD [6]. Besides, our previous study has shown that *IL5RA* variants are significantly associated with COPD susceptibility [7]. The harm of COPD is not limited to the respiratory system but also may increase the risk of complications such as cardiovascular disease, osteoporosis, depression, and anxiety [2,8]. Therefore, an in-depth study on the etiology, pathogenesis, and genetic factors of COPD is of great significance for developing new prevention and treatment strategies.

RARB (retinoic acid receptor beta), a member of the retinoic acid receptor family, is a nuclear receptor that plays a pivotal role in regulating cell growth, differentiation, and apoptosis across various cell types. Its ability to bind to retinoic acid and modulate the expression of specific genes positions *RARB* as a key player in both physiological and pathological processes. Studies have shown that the expression level of *RARB* is closely related to the occurrence and development of COPD. For instance, Wu et al. showed that the variation of the *RARB* gene interacts with polycyclic aromatic hydrocarbon exposure, thus affecting the annual changes in lung function, which indicates that *RARB* may play a key role in the interaction between environmental factors and genetic factors of COPD [9]. In addition, Pelos G et al. pointed out the sensitivity of non-small cell lung cancer (NSCLC) cells to demethylation drugs and retinoic acid, which further confirmed the importance of *RARB* in lung diseases [10]. Beyond its implications in lung diseases, *RARB*’s abnormal expression has also been implicated in the development of various diseases, including certain cancers. For example, the abnormal expression of *RARB* may lead to uncontrolled cell proliferation and differentiation disorders [11]. Notably, it was found that the fusion gene *FNDC3B*::*RARB* was related to all-trans retinoic acid resistance in acute promyelocytic leukemia (APL), which indicated the potential role of *RARB* in leukemia treatment [12]. In summary, *RARB* genetic variants hold promise as biomarkers or therapeutic targets in COPD. However, the correlation between *RARB* gene polymorphism and susceptibility to COPD is unclear.

Here, we conducted a case-control study to determine the roles of *RARB* gene polymorphisms in COPD risk among the Chinese Han population. The purpose of this study is to reveal the potential association between *RARB* gene variations and the risk of developing COPD and to provide new directions and strategies for early identification, preventive measures, and individualized treatment plans for the disease. Our research will not only reveal the specific role of *RARB* gene variation in the susceptibility of COPD but also provide new directions and strategies for early identification, preventive measures, and personalized treatment plans. Through these findings, we hope to offer more precise medical interventions for COPD patients, thereby improving treatment outcomes, reducing the disease burden, and ultimately advancing the progression of COPD treatment and prevention strategies.

## Materials and methods

### Study population

We determined the sample size using G* Power (version 3.1.9.7) software. Initially, the statistical method chosen was the *t*-test, followed by selecting the classification for ‘Difference between two independent means (two groups)’. The parameters were then set as follows: tail = 2, effective size = 0.26, α = 0.05, power = 0.85, and allocation ratio = 1. Finally, the sample size for both the case group and the control group was calculated to be 267 cases each. In this case-control study, a total of 541 participants, including 270 patients with COPD and 271 healthy controls, were recruited at Hainan General Hospital. This study was approved by the ethics committee of Hainan General Hospital (Medical Ethics Research [2022] No. 312) and was conducted based on the 1964 Declaration of Helsinki’s ethical standards. Before the study, all subjects were informed of the purpose of the study and signed the written informed consent form. An individual was deemed to have COPD if they exhibited common respiratory symptoms including persistent cough, expectoration of sputum, breathing difficulty, and wheezing during their everyday activities following the guidelines by the Global Initiative for Chronic Obstructive Pulmonary Disease 2023 [13]. Additionally, the diagnosis was confirmed by a post-bronchodilator FEV1/FVC ratio that fell below the threshold of 70%, observed 30 min after the administration of 400 µg of salbutamol sulfate. The inclusion criteria were: 1) age> 18 years old. 2) Individuals of Chinese Han ethnicity who have resided in the Hainan region for a minimum of three generations without intermarriage with other ethnic groups. 3) Participants must exhibit sound mental health and possess the cognitive capacity to complete the survey. 4) They must be aware of the study’s objectives and have consented to participate voluntarily. The exclusion criteria were: 1) Individuals of non-Han descent or those with a history of intermarriage with other ethnic groups within the past three generations. 2) Presence of restrictive ventilatory disorders, such as active tuberculosis, chest deformities, concurrent pleural effusions, or bronchopulmonary cancer. 3) Conditions that result in obstructive ventilatory issues, including tuberculosis-induced lung disfigurement and bronchiectasis. 4) any medical history of lung diseases, such as lung cancer and bronchial asthma. The control group was selected from healthy individuals who had a physical examination in the same hospital, with normal lung function, and matched with the gender and age of the case. Demographic data (such as age, gender, BMI, smoking status, GOLD severity stage, age of smoking onset, years smoked, and comorbidities) and clinical indicators were obtained through medical records and standard questionnaires.

### SNP selection and genotyping

The selection criteria for SNPs were concisely defined as follows: Firstly, SNPs had to exhibit a minor allele frequency (MAF) exceeding 5% among Chinese Han individuals as documented in the 1000 Genomes Project. Secondly, they were required to be in low linkage disequilibrium with one another, with an R^2^ value below 0.8. Thirdly, the minimum genotype frequency had to be above 75%. Fourthly, they had to comply with Hardy-Weinberg equilibrium (HWE), with a *p*-value greater than 0.05. Lastly, the call rate for each SNP needed to be over 95%. Based on these criteria, six SNPs (rs6799734 G > C, rs1529672 C > A, rs1286655 A > C, rs1286641 T > A, rs1298216 A > G, and rs1881706 G > A) were identified as fitting the specified requirements. Genomic DNA was extracted from the peripheral blood samples of the subjects with a Gold Mag-Mini Purification Kit (GoldMag Co. Ltd., Xi’an, China). The extracted DNA was frozen in an EDTA test tube. DNA concentration was detected by NanoDrop 2000 (Thermo Scientific, Waltham, USA), and SNP genotyping was performed by the Agena MassARRAY iPLEX platform (Agena Bioscience Inc., CA, USA) [14]. Genotyping data were analyzed by Agena Typer software (Version 4.0). The primer sequences of PCR amplification are shown in Table S1.

### Bioinformatic analysis

HaploReg v4.2 online software (https://pubs.broadinstitute.org/mammals/haploreg/haploreg.php) was performed to predict the potential function of the six SNPs [15]. Besides, we evaluated the association between the SNPs and *RARB* expression based on the expression quantitative trait loci (eQTL) analysis *via* the Genotype-Tissue Expression (GTEx) database (http://www.gtexportal.org/) [16].

### Statistical analysis

Two-sided *χ^2^* test or independent sample *t-*tests were used to analyze the differences in the distribution of demographic and clinical variables between the case group and the control group. An extract test was used to evaluate whether the SNP genotypes in the control group conformed to HWE. Logistic regression analysis of multiple genetic models was used to evaluate the correlation between each SNP and COPD risk, which provided adjusted odds ratios (ORs) with 95% confidence intervals (CIs), adjusting for age, gender, body mass index (BMI), and smoking. Further estimations of associations were conducted within subgroups categorized by age, gender, BMI, and smoking status, adjusting for age, gender, BMI, and smoking. The Benjamini and Hochberg’s false discovery rate (FDR) method was used to correct for multiple testing corrections. The false positive reporting probability (FPRP) analysis was used to evaluate the noteworthy association [17]. This method combines the observed effect size (such as OR), the CI of the effect size, prior probability, and the levels of significance to calculate a probability value, indicating the likelihood that the results are false positives under given conditions, The lower the FPRP value, the more likely the results are to be true. Finally, we analyzed the effect of SNP-SNP interaction on the risk of COPD by multivariate dimensionality reduction (MDR). A dendrogram was prepared to show the interaction of individual factors in the best predictive model through information gain values (entropy percentage). Statistical analyses were performed using SPSS 22.0 software, with all tests conducted as two-tailed. A *p*-value of less than 0.05 was set as the threshold for statistical significance.

## Results

### Demographic characteristics

The details of the participants are presented in Table 1. The study involved 270 Chinese Han COPD patients, comprising 64 females and 206 males, and 271 healthy Chinese Han individuals, with 61 females and 210 males. The average age of the case group was 72.26 ± 10.40 years old, which was significantly higher than that of the control group (69.21 ± 6.61 years old), with the *p*-value less than 0.001. However, no significant differences were observed between the two groups in terms of gender, smoking status, and BMI, with all *p*-values exceeding 0.05.

**Table 1.** Basic characteristics of the study participants.

Characteristics	COPD (*N* = 270)	Controls (*N* = 271)	*p*
Age, year	72.26 ± 10.40	69.21 ± 6.61	< 0.001^a^
≤ 71	108 (40.0%)	180 (66.4%)	
> 71	162 (60.0%)	91 (33.6%)	
Sex			0.742^b^
Male	206 (76.3%)	210 (77.5%)	
Female	64 (23.7%)	61 (22.5%)	
Smoking status			0.518^b^
Yes	142 (52.6%)	135 (49.8%)	
No	128 (47.4%)	136 (50.2%)	
Age of smoking onset (years)	23.1 ± 8.23		
Years smoked (years)	37.5 ± 13.19		
Smoking (current/former/never/unclear), n	51/109/104/6		
BMI (kg/m^2^)			0.424^b^
≥ 24	57 (21.1%)	65 (24.0%)	
< 24	213 (78.9%)	206 (76.0%)	
Respiratory rate (times/minute)	22.29 ± 2.44		
FVC (L)	4.92 ± 32.99		
FEV1 (L)	1.46 ± 3.06		
FEV1/FVC (%)	39.73 ± 29.42		
GOLD I, n (%)	238 (88.1%)		
GOLD II, n (%)	20 (7.4%)		
GOLD III, n (%)	12 (4.5%)		
Comorbidities (yes/no/unavailable), n	86/164/20		

COPD: Chronic obstructive pulmonary disease; BMI: body mass index; FVC: forced vital capacity; FEV1: forced the first second of expiratory volume. a. *p*-value was evaluated by *t*-test. b. *p-*value was tested by a two-sided χ^2^ test. *p* < 0.05 presents a significant difference.

### Analysis of the association between *RARB* polymorphisms and COPD risk

**Figure 1.**
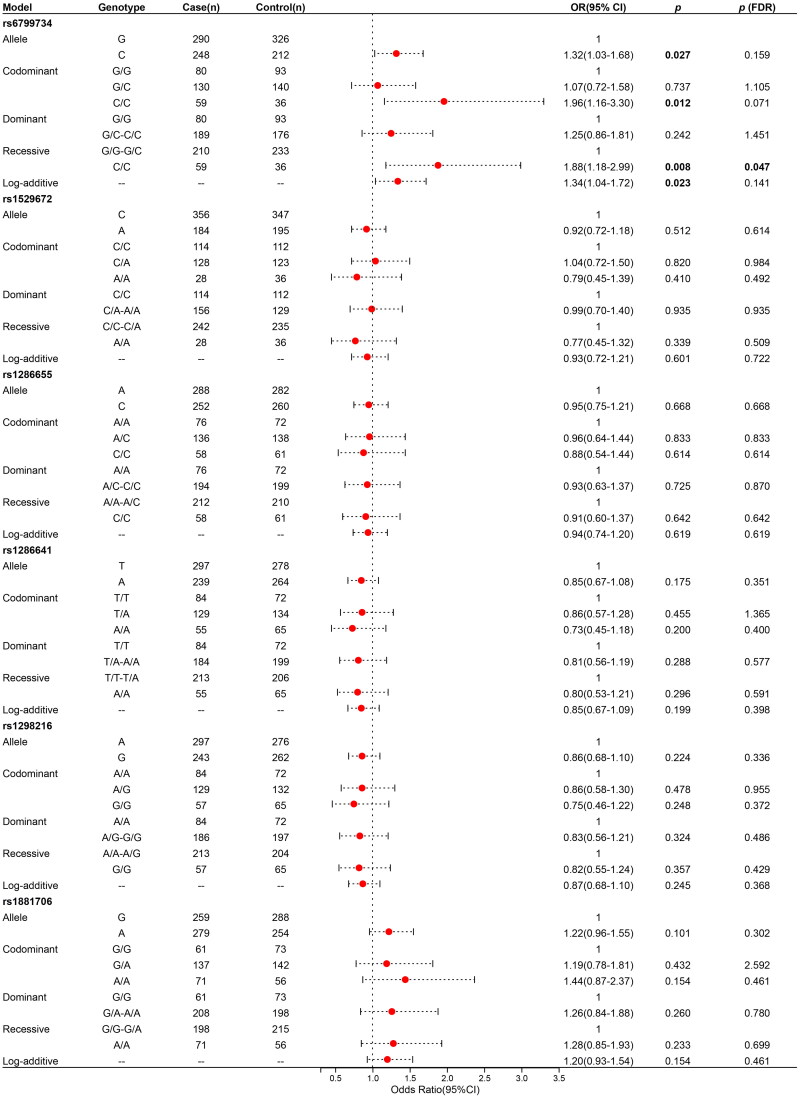
The relationship between *RARB* polymorphisms and COPD susceptibility COPD, chronic obstructive pulmonary disease; SNP, single nucleotide polymorphisms; or, odds ratios; 95% CI, 95% confidence interval; FDR, false discovery rate. The *p*-value was calculated by logistic regression analysis with adjustment for age, gender, body mass index, and smoking. *p* < 0.05 represents statistically significant. The threshold value for FDR was 0.05.

This study successfully genotyped six SNPs (rs6799734, rs1529672, rs1286655, rs1286641, rs1298216, and rs1881706) in the *RARB* gene. As shown in Table 2, the MAF of these SNPs was greater than 0.05 in the case and control group. In the control group, all of these SNPs followed HWE (*p* > 0.05). The association of these SNPs with the risk of COPD is depicted in Figure 1. The rs6799734 was significantly associated with the risk of COPD under the allele (C Vs G, OR 1.32, 95% CI = 1.03-1.68, *p* = 0.027, *p* (FDR) = 0.159), codominant (CC Vs GG, OR 1.96, 95% CI = 1.16-3.30, *p* = 0.012, *p* (FDR) = 0.071), recessive (CC Vs GG-GC, OR 1.88, 95% CI = 1.18-2.99, *p* = 0.008, *p* (FDR) = 0.047), and log-additive (OR 1.34, 95% CI = 1.04-1.72, *p* = 0.023, *p* (FDR) = 0.141) models.

**Table 2.** Basic information about six SNPs identified in the *RARB* gene.

SNP	Region	Position	Allele (A/B)	MAF		HWE *p*	HaploReg v4.2
				Case	Control		
rs6799734	Intron	3: 25444320	C/G	0.461	0.394	0.161	Enhancer histone marks, Motifs changed
rs1529672	Intron	3: 25479091	A/C	0.341	0.360	0.793	Enhancer histone marks, DNAse, Motifs changed, NHGR1/EBI GWAS hits, GRASP QTL hits
rs1286655	Intron	3: 25482061	C/A	0.467	0.480	0.808	Enhancer histone marks, Motifs changed
rs1286641	Intron	3: 25502379	A/T	0.446	0.487	0.903	Enhancer histone marks, DNAse, Proteins bound, Motifs changed
rs1298216	Intron	3: 25504885	G/A	0.450	0.487	0.807	Enhancer histone marks, Motifs changed
rs1881706	Intron	3: 25508961	A/G	0.519	0.469	0.464	Enhancer histone marks, DNAse, Proteins bound, Motifs changed, GRASP QTL hits

SNP: single nucleotide polymorphism; A: minor; B: major; MAF: minor allele frequency; HWE: Hardy-Weinberg equilibrium.

### Stratification analysis of the *RARB* polymorphisms and COPD risk

We conducted a further analysis to evaluate the relationship between *RARB* SNPs and COPD susceptibility, stratified by age, gender, BMI, and smoking status, as detailed in Table 3. The result showed that rs6799734 was linked with increased susceptibility to COPD among individuals aged ≤ 71 years (CC Vs GG, OR 2.20, *p* = 0.037, *p* (FDR) = 0.220; CC Vs GG-GC, OR 2.34, *p* = 0.011, *p* (FDR) = 0.045), males (C Vs G, OR 1.45, *p* = 0.008, *p* (FDR) = 0.049; CC Vs GG, OR 2.60, *p* = 0.002, *p* (FDR) = 0.013; CC Vs GG-GC, OR 2.35, *p* = 0.002, *p* (FDR) = 0.013), those with BMI ≥ 24 (CC Vs GG-GC, OR 3.95, *p* = 0.018, *p* (FDR) = 0.108), and smokers (CC Vs GG, OR 2.48, *p* = 0.020, *p* (FDR) = 0.120; CC Vs GG-GC, OR 2.29, *p* = 0.016, *p* (FDR) = 0.095). Additionally, the SNPs rs1286641 and rs1881706 demonstrated a significant risk correlation with COPD in females (rs1286641: A Vs T, OR 1.73, *p* = 0.032, *p* (FDR) = 0.193; AA Vs TT, OR 2.89, *p* = 0.049, *p* (FDR) = 0.293; TA-AA Vs TT, OR 2.65, *p* = 0.031, *p* (FDR) = 0.187; rs1881706: GA Vs GG, OR 2.59, *p* = 0.039, *p* (FDR) = 0.234) and smokers (rs1286641: A Vs T, OR 1.41, *p* = 0.044, *p* (FDR) = 0.131; rs1881706: A Vs G, OR 1.42, *p* = 0.040, *p* (FDR) = 0.243).

**Table 3.** Stratification analyses of *RARB* polymorphisms with COPD susceptibility.

	Model	Allele/genotype	Adjusted OR (95% CI)	*p*	*p* (FDR)
Age ≤ 71 years					
rs6799734	codominant	G/G	1.00 (reference)		
		C/C	2.20 (1.05-4.62)	**0.037**	0.220
	recessive	G/G-G/C	1.00 (reference)		
		C/C	2.34 (1.22-4.50)	**0.011**	**0.045**
Male					
rs6799734	Allele	G	1.00 (reference)		
		C	1.45 (1.10-1.91)	**0.008**	**0.049**
	codominant	G/G	1.00 (reference)		
		C/C	2.60 (1.41-4.80)	**0.002**	**0.013**
	recessive	G/G-G/C	1.00 (reference)		
		C/C	2.35 (1.36-4.06)	**0.002**	**0.013**
	Log-additive	–	1.53 (1.14-2.06)	**0.005**	**0.028**
Female					
rs1286641	allele	T	1.00 (reference)		
		A	1.73 (1.05-2.85)	**0.032**	0.193
	codominant	T/T	1.00 (reference)		
		A/A	2.89 (1.01-8.29)	**0.049**	0.293
	dominant	T/T	1.00 (reference)		
		T/A-A/A	2.65 (1.09-6.44)	**0.031**	0.187
rs1881706	codominant	G/G	1.00 (reference)		
		G/A	2.59 (1.05-6.37)	**0.039**	0.234
BMI ≥ 24					
rs6799734	recessive	G/G-G/C	1.00 (reference)		
		C/C	3.95 (1.27-12.35)	**0.018**	0.108
Smoking status					
rs1286641	allele	T	1.00 (reference)		
		A	1.41 (1.01-1.98)	**0.044**	0.131
rs1881706	allele	G	1.00 (reference)		
		A	1.42 (1.02-1.98)	**0.040**	0.243
rs6799734	codominant	G/G	1.00 (reference)		
		C/C	2.48 (1.15-5.33)	**0.020**	0.120
	recessive	G/G-G/C	1.00 (reference)		
		C/C	2.29 (1.17-4.48)	**0.016**	0.095
	Log-additive	–	1.50 (1.04-2.17)	**0.032**	0.189

SNP: single nucleotide polymorphism; OR: odds ratio; CI: confidence interval.

The *p*-value was calculated by logistic regression analysis with adjustment for age, gender, body mass index, and smoking. Bold text represents statistical significance.

The threshold value for FDR was 0.05.

### FPRP result

The above significant findings were further evaluated by the analysis of FPRP (Table 4). When the prior probability is 0.25 and the FPRP threshold is set to 0.2, the association between rs6799734 and COPD risk is still worthy of attention in the overall and stratified results, except in the stratification with BMI ≥ 24. In the stratification results of women and smoking status, rs1286641 also found noteworthy results. Moreover, the findings for rs1881706 within the smoking status stratification remain statistically significant.

**Table 4.** False-positive report probability analysis for the positive findings.

Genotype	OR (95 % CI)	*p* ^a^	Statistical Power^b^	Prior Probability				
				0.25	0.1	0.01	0.001	0.0001
rs6799734 G > C								
C Vs G	1.32(1.03-1.68)	0.027	1.000	0.067^c^	0.178^c^	0.704	0.960	0.996
C/C Vs G/G	1.96(1.16-3.30)	0.012	0.530	0.060^c^	0.162^c^	0.679	0.955	0.997
C/C Vs G/G-G/C	1.88(1.18-2.99)	0.008	0.603	0.037^c^	0.103^c^	0.557	0.927	0.998
Age ≤ 71 years								
rs6799734 G > C								
C/C Vs G/G	2.20 (1.05-4.62)	0.037	0.401	0.218	0.456	0.902	0.989	0.985
C/C Vs G/G-G/C	2.34 (1.22-4.50)	0.011	0.319	0.092^c^	0.234	0.771	0.971	0.997
Male								
rs6799734 G > C								
C Vs G	1.45 (1.10-1.91)	0.008	0.989	0.024^c^	0.070^c^	0.451	0.892	
C/C Vs G/G	2.60 (1.41-4.80)	0.002	0.201	0.033^c^	0.092^c^	0.526	0.918	0.994
C/C Vs G/G-G/C	2.35 (1.36-4.06)	0.002	0.282	0.023^c^	0.065^c^	0.435	0.886	0.996
Female								
rs1286641 T > A								
A Vs T	1.73 (1.05-2.85)	0.032	0.715	0.116^c^	0.283	0.813	0.978	
A/A Vs T/T	2.89 (1.01-8.29)	0.049	0.247	0.370	0.638	0.951	0.995	0.998
T/T-T/A Vs T/T	2.65 (1.09-6.44)	0.031	0.267	0.261	0.514	0.921	0.992	0.998
rs1881706 G > A								
GA Vs GG	2.59 (1.05-6.37)	0.039	0.287	0.286	0.545	0.930	0.993	0.995
BMI ≥ 24								
rs6799734 G > C								
C/C Vs G/G-G/C	3.95 (1.27-12.35)	0.018	0.121	0.311	0.575	0.937	0.993	0.997
Smoking status								
rs1286641 T > A								
A Vs T	1.41 (1.01-1.98)	0.044	0.978	0.127^c^	0.303	0.827	0.980	0.998
rs1881706 G > A								
A Vs G	1.42 (1.02-1.98)	0.040	0.978	0.106^c^	0.263	0.797	0.975	0.996
rs6799734 G > C								
C/C Vs G/G	2.48 (1.15-5.33)	0.020	0.291	0.171^c^	0.382	0.872	0.986	0.993
C/C Vs G/G-G/C	2.29 (1.17-4.48)	0.016	0.346	0.119^c^	0.287	0.816	0.978	0.976

p value ^a^ was calculated by unconditional logistic regression analysis with adjustment for age, gender, body mass index, and smoking.

Statistical power ^b^ was calculated using the number of observations in the subgroup and the OR and p values in this table.

^c^ The level of false-positive report probability threshold was set at 0.2 and noteworthy findings are presented.

### SNP-SNP interaction analysis and COPD susceptibility

**Figure 2.**
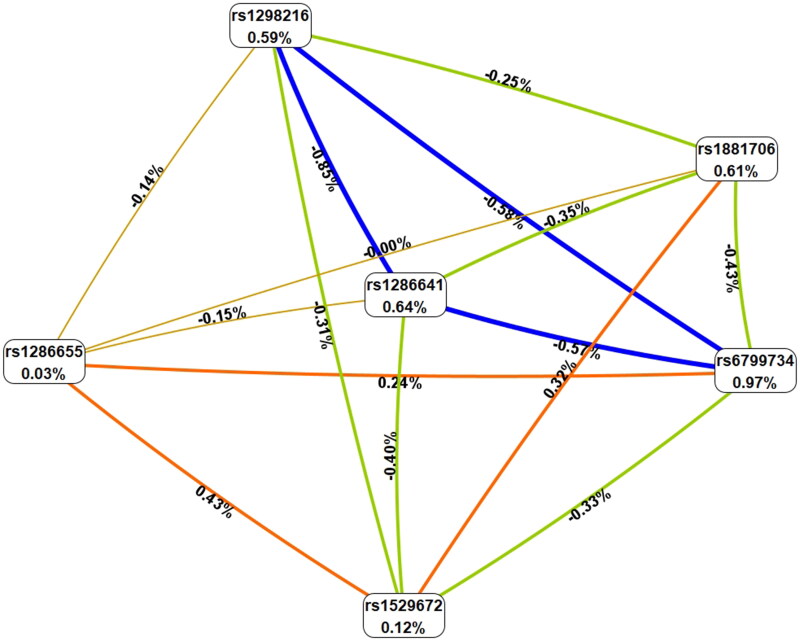
Tree diagram analysis of SNP interactions. The interaction model describes the percentage of the entropy (information gain) that is explained by each factor. Values inside nodes indicate information gain of individual attributes or main effects, whereas values between nodes show information gain of pairwise combinations of attributes or interaction effects. Positive entropy (plotted in orange) indicates interaction, which can be interpreted as a synergistic or nonadditive relationship; while negative entropy (plotted in green or blue) indicates independence or additivity (redundancy). Information gain is automatically calculated by the MDR software. The software evaluates information gain by comparing the accuracy of different genetic marker combinations in classifying disease status. Main effects refer to the independent impact of individual genetic markers on disease risk.

MDR analysis was used to study the effect of SNP-SNP interaction on the risk of COPD, and the results are shown in Table 5 and Figure 2. As shown in Table 5, the combination of rs6799734, rs1529672, rs1286655, rs1286641, rs1298216, and rs1881706 was the best prediction model for COPD (OR 3.97, 95% CI 2.78-5.70, *p* < 0.0001) with the perfect CVC (10/10). The interaction model describes the percentage of the entropy (information gain) that is explained by each factor or 2-way interaction. Values inside nodes indicate information gain of individual attributes or main effects, whereas values between nodes show information gain of pairwise combinations of attributes or interaction effects. Positive entropy (plotted in orange) indicates interaction, which can be interpreted as a synergistic or nonadditive relationship; while negative entropy (plotted in blue or green) indicates independence or additivity (redundancy). The interaction diagram shows the following interaction order: rs1286655 × rs1529672 > rs1529672 × rs1881706 > rs1286655 × rs6799734, which has higher positive entropy or synergy (0.43%, 0.32%, and 0.24% respectively, shown in orange). Low entropy means redundancy or even independence (Figure 2).

**Table 5.** SNP-SNP interaction analysis in *RARB* by MDR.

Model	Testing Bal. Acc.	CVC	OR (95% CI)	*p*
rs6799734	0.5222	9/10	1.82 (1.15-2.86)	0.009
rs6799734,rs1286641	0.4537	3/10	1.63 (1.15-2.29)	0.005
rs6799734,rs1286641,rs1881706	0.4981	4/10	2.18 (1.54-3.08)	< 0.0001
rs6799734,rs1529672,rs1298216,rs1881706	0.5241	6/10	3.05 (2.14-4.35)	< 0.0001
rs6799734,rs1529672,rs1286655,rs1286641,rs1881706	0.5111	8/10	3.71 (2.59-5.29)	< 0.0001
rs6799734,rs1529672,rs1286655,rs1286641,rs1298216,rs1881706	0.5167	10/10	3.97 (2.78-5.70)	< 0.0001

MDR: multifactor dimensionality reduction; Bal. Acc.: balanced accuracy; CVC: cross-validation consistency; OR: odds ratio; 95% CI: 95% confidence interval.

*p* values were calculated by χ^2^ test.

### Impact of SNPs on gene expression (eQTLs)

**Figure 3.**
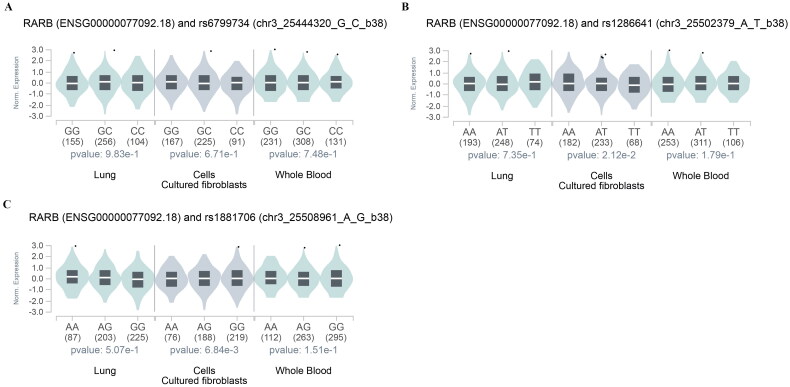
GTEx Analysis for the association between rs6799734 (a), rs1286641 (B), and rs1881706 (C) and *RARB* gene expression.

We further analyzed the expression loci (eQTLs) of rs6799734, rs1286641, and rs1881706 by GTEx. No significant correlation was observed between rs6799734 genotypes and *RARB* expression level (Figure 3A). Interestingly, rs1286641 (Figure 3B) and rs1881706 (Figure 3C) were significantly correlated with the expression of *RARB* mRNA in cells-cultured fibroblasts (*p* = 2.12 × 10^−2^ and *p* = 6.84 × 10^−3^, respectively).

## Discussion

COPD is a progressive respiratory system disease, which seriously endangers the respiratory function and quality of life of patients and may lead to death [18]. Genetic factors play an important role in the pathogenesis of COPD [19,20]. Previous studies have identified some susceptibility gene loci related to lung function, especially the *RARB* gene [21–23]. However, there is no report to confirm the relationship between genetic polymorphism of the *RARB* gene and the risk of COPD. We conducted the first study to investigate the relationship between genetic variants in the *RARB* gene and the risk of COPD. Our findings indicate that variants within the *RARB* gene are significantly linked to an increased susceptibility to COPD. This research could help elucidate the biological and genetic mechanisms underlying COPD.

Our study indicated that rs6799734 was significantly associated with an increased susceptibility to COPD. On the contrary, Tran et al. showed that rs6799734 was correlated with a decreased susceptibility to meningomyelocele [24]. The difference between COPD and myelomeningocele may be due to its pleiotropic effects in different diseases through unique biological pathways, genetic background, environmental factors, and regulatory mechanisms. Multiple genetic loci associated with COPD have been identified through GWAS, such as *CHRNA4*, *AFAP1*, and *DTWD1* [25]. We can more accurately identify high-risk individuals through integrative analysis of multiple genetic markers, thereby enabling early intervention and preventive measures. Besides, some researchers identified SNP loci associated with peripheral blood protein concentrations by integrating GWAS and proteomics data, such as SNPs in the *AGER* gene, which may serve as biomarkers for emphysema in COPD [26]. These findings suggest that rs6799734 along with these genetic markers could serve as potential genetic markers for COPD risk assessment, which may contribute to early diagnosis, personalized treatment strategies, and preventive measures for COPD patients. However, these results are preliminary, and further research, including larger studies and functional analyses, is necessary to validate the clinical utility of these SNPs in COPD diagnosis. Besides, a study found that *RARB* rs1579672 was significantly associated with an increased risk of COPD [27]. However, we did not observe a significant association between rs1579672 and COPD. The main reason for this may be the difference in the people studied. Our study involved a Chinese Han population, whereas the referenced literature utilized a European population. Additionally, our research adopted a case-control study, while the literature adopted a meta-analysis method. Other factors, such as sample size and quality control measures, could also lead to differences in observation results. Besides, no significant associations were observed between rs1286655, rs1298216, and COPD susceptibility. These findings suggest that rs1529672, rs1286655, and rs1298216 may not play a significant role in the development of COPD. Age, gender, BMI, and smoking consumption significantly affect the risk of COPD [28–30]. We conducted a stratified analysis of *RARB* gene polymorphisms with COPD risk, considering age, gender, BMI, and smoking status. We found that rs6799734 was linked with increased susceptibility to COPD among individuals aged ≤ 71 years, males, those with BMI ≥ 24, and smokers. This suggests that the effect of this SNP may be more pronounced in younger individuals and those with higher BMI. Additionally, the SNPs rs1286641 and rs1881706 demonstrated a significant risk correlation with COPD in females and smokers, indicating that gender and smoking status may modulate the genetic susceptibility to COPD. Similar to our results, Fu et al. showed that rs2201584 in the IL12Rβ2 was remarkably related to an increase in the risk of COPD in subjects aged ≤ 68 years and females, but not in those aged > 68 years and males [31]. Besides, LINC01414 rs298207 could increase susceptibility to COPD in males but not in females. However, LINC00824 rs7815944 had a protective role in males, but not in females [32]. MIR2113 rs2505059 could decrease the risk of COPD in subjects aged > 70 years, male, and non-smokers [33]. Additionally, the CHRNA3/5 locus was linked to lung function in individuals with heavy smoking habits [34]. Among nonsmokers, rs7934083 and rs4719841 emerged as protective factors against COPD susceptibility [35]. In addition, *SREK1* rs74794265 exhibited a significant association with COPD risk, whereas this link was not observed in smokers [36]. These findings further emphasize the importance of considering environmental factors, such as smoking, in the genetic study of COPD. Wu et al. provide compelling evidence that there is a significant interaction between *RARB* variants and exposure to polycyclic aromatic hydrocarbons (PAHs), which significantly affects the annual changes in lung function of COPD patients [9]. Our findings and the results of Wu et al. further emphasize the importance of considering environmental factors in the genetic study of COPD. Based on these findings, we infer that genetic susceptibility to COPD may be regulated by age, gender, and BMI, which highlights the importance of paying attention to individual heterogeneity when exploring the relationship between genetic factors and COPD risk. In conclusion, our stratified analysis results indicate that age, gender, BMI, and smoking status play a crucial role in the relationship between genetic variants and COPD risk. Future studies should further explore how these factors modulate the genetic susceptibility to COPD and consider the impact of environmental factors

The SNPs rs6799734, rs1286641, and rs1881706 are located within the intronic region of the *RARB* gene. While there are currently no specific studies addressing these SNPs, existing research indicates that genetic variations within introns can influence gene expression levels by altering splicing sites, mRNA stability, or creating/destroying binding sites for transcription factors [37–39]. Here, we assessed the impact of rs6799734, rs1286641, and rs1881706 on the expression levels of the *RARB* gene utilizing the GTEx database. We observed that rs1286641 and rs1881706 were significantly correlated with the expression of *RARB* mRNA in cells-cultured fibroblasts. Additionally, we performed HaploReg v4.2 online software to explore the potential functions of these SNPs. We found that rs6799734 was associated with the regulation of Enhancer histone marks and Motifs changed. Rs1286641 could influence the regulation of Enhancer histone marks, DNAse, Proteins bound, and Motifs changed. And rs1881706 was related to Enhancer histone marks, DNAse, Proteins bound, Motifs changed, and GRASP QTL hits. Given that *RARB* is pivotal in regulating inflammatory responses, cell proliferation, and apoptosis, we hypothesize that these intronic SNPs may modulate *RARB* expression and thereby impact cellular processes critical to the pathogenesis of COPD [9]. Specifically, we propose that these SNPs could affect the interaction between transcription factors and regulatory elements associated with the RARB gene, leading to alterations in gene expression and downstream signaling pathways. This modulation could potentially influence the risk of developing COPD by affecting essential cellular processes such as signal transduction, proliferation, differentiation, or apoptosis. Future investigations will aim to validate the functional implications of these SNPs on RARB expression and their association with COPD risk.

Our study has some limitations. Firstly, Although the sample size is statistically sufficient, it is relatively small for genetic association studies, which may limit our ability to detect associations, especially for rare variants or complex traits. Future work needs to expand the sample size to verify our findings. Secondly, the study participants were limited to individuals of Han Chinese ethnicity, which restricts the generalizability of our findings to other ethnic populations. Different ethnic groups exhibit varying frequencies of genetic polymorphisms, which can be attributed to factors such as genetic drift, natural selection, and migration patterns. For instance, Gim et al. conducted a study comparing the genetic risk of COPD across different ethnicities, highlighting the significance of racial differences in genetic predispositions [40]. Environmental factors, including smoking, pollution, and diet, can significantly influence the expression and impact of genetic polymorphisms. A study by Park et al. explored gene-smoking interactions in COPD, demonstrating how environmental exposures can modulate genetic outcomes [41]. Overall, the influence of different races and environmental factors on genetic polymorphisms is both complex and profound. Racial disparities can result in varying frequencies of genetic polymorphisms across populations, while environmental factors, such as smoking, can interact with genetic elements to further impact disease risk and presentation. These insights are crucial for comprehending the genetic underpinnings of diseases, devising personalized medical approaches, and enhancing public health outcomes. Future studies should delve deeper into the interplay between genetic polymorphisms and environmental factors within diverse racial groups, aiming to offer more precise targets for disease prevention and treatment. Additionally, due to a lack of relevant data, we were unable to examine the impact of these SNPs on clinical outcomes. We intend to collect more clinical data in future studies to evaluate the influence of these gene mutations on disease progression and treatment response. Finally, to fully understand the implications of the *RARB* gene and its significant SNPs, it is necessary to conduct functional analyses through *in vitro* and *in vivo* experiments. We plan to incorporate these analyses in future research to explore the biological functions and potential molecular mechanisms of these genetic variations. Despite these advantages, our study first identified the role of *RARB* gene variation in COPD susceptibility. Our findings not only provide new insights into the genetic basis of COPD but also have potential broad implications for future research directions, prevention strategies, and personalized treatment options for COPD patients. Firstly, our discoveries can guide future research by exploring the role of other genetic variants or biomarkers in COPD, further deepening our understanding of the disease mechanisms. Secondly, our research results can inform the development of prevention strategies targeted at specific genetic risk groups. For instance, by identifying high-risk individuals through genetic testing, we can provide more targeted preventive measures for them. Additionally, our study paves the way for the development of personalized treatment plans, such as tailoring therapeutic approaches based on the genetic background of the patients. Lastly, our research findings may also impact clinical practice, assisting healthcare providers in more accurately assessing and managing COPD patients.

## Conclusion

In summary, our study highlighted the significant impact of *RARB* gene SNPs on COPD risk. These insights could aid in the creation of screening tools for genetic counseling and enhance our comprehension of how genetic variants contribute to COPD susceptibility.

## Supplementary Material

supplement material.docx

## Data Availability

The data that support the findings of this study are available from the corresponding author upon reasonable request.
