# Supplementary material for: RARB genetic variants might contribute to the risk of chronic obstructive pulmonary disease based on a case-control study
Source: Ann Med. 2024 Dec 26;57(1):2445195. doi: 10.1080/07853890.2024.2445195 (PMC11703482; doi:10.1080/07853890.2024.2445195)
Supplement: supplement material.docx [file IANN_A_2445195_SM3833.docx]

Table S1 Primer information for the RARB SNPs in this study

| SNP | 1^st^-PCR primer | 2^nd^-PCR primer | UEP_SEQ |
| --- | --- | --- | --- |
| rs6799734 | ACGTTGGATGGCTTCTAATCTTCCCCATCC | ACGTTGGATGGTGAGTCCAAAGTTCCCAAC | aTTTGCCAACGATATAAATTTCTATTTT |
| rs1529672 | ACGTTGGATGGGGCCTTTAATTGTACCCTC | ACGTTGGATGTTAAAACAGCCCAGCCTTAG | aatCCTTAGAAATATGTACAGAATTCAT |
| rs1286655 | ACGTTGGATGGCAGAGGCTTCTTATTGCAG | ACGTTGGATGGAACTTGAACCTAGGGTATC | ggataTGGCAGCAGGACCCAAGCCC |
| rs1286641 | ACGTTGGATGTGAGCACCCTCAGAGTAATG | ACGTTGGATGTTGGAACTGGGAAACGTGAC | AGGTAAAGCCCCTGT |
| rs1298216 | ACGTTGGATGAAGCGATACTCCTGCCTAAG | ACGTTGGATGTGGCCAACTTGATGAAACGC | GGCGCCTATAATGCCAGCTA |
| rs1881706 | ACGTTGGATGTACTTCCTTTTCAGTCTGAG | ACGTTGGATGTTATGTTTGGGTACCAGCAG | ggGCCACTGTGGGACTCAA |

SNP, single nucleotide polymorphisms; PCR, polymerase chain reaction; UEP_SEQ: unextended mini-sequencing primer.
